# Photo-Documentation of Thumbnail Regrowth After Surgical Avulsion: Case Report and Literature Review

**Published:** 2014-07-09

**Authors:** Ashley Marie Mefford, Morton L. Kasdan, Bradon Wilhelmi

**Affiliations:** ^a^University of Louisville School of Medicine; ^b^Robley Rex Veterans Affairs Medical Center, Department of Surgery, University of Louisville School of Medicine; ^c^Division of Plastic and Reconstructive Surgery, Department of Surgery, University of Louisville School of Medicine, Louisville, Ky.

**Keywords:** avulsion, growth rate, regrowth, thumbnail, time-lapse video

## Abstract

**Objective:** We present a case of thumbnail regrowth and photo-documentation after a complete surgical avulsion and nail bed biopsy. **Methods:** A complete surgical avulsion of the right thumbnail was performed and the regrowth was photo-documented. **Results:** Complete regrowth was achieved by 33 weeks after avulsion, and the photos were compiled into a time-lapse video. **Conclusions:** The established literature indicates that rate of nail growth is multifactorial. One variable that may contribute to rate of growth is the status of the nail itself, intact or avulsed. The growth rate of the thumbnail in our patient is comparable to the growth rates documented for intact nails. The literature on this subject is reviewed.

Human fingernails are an important piece of digit anatomy. They are thought of as cosmetic. They contribute to the function of the digit through tactile sensation, thermoregulation, and protection.^1^^,^^2^ Their functional importance and superficial location have made them a subject of study and publication for more than 100 years. While several papers have discussed the growth of nails and the influencing factors, relatively few discuss the regrowth of nails after surgical avulsion. One study by Lai et al reports on the outcomes of 33 nail avulsion procedures in subjects 27 to 86 years of age.^3^ The reported time to full regrowth ranged from 5 to 10 months, including fingernails and toenails. In this report, we contribute a 9-month photo record of the regrowth of a thumbnail following a surgical avulsion.

## METHODS/CASE PRESENTATION

The case we present is that of a 76-year-old, nonsmoking man. The patient was a surgeon whose hobbies included woodworking. A nail deformity developed in his right thumbnail. The deformity was a tender, dark-yellow, longitudinal strip reaching the entire length of the nail bed. A complete nail avulsion and nail bed biopsy were completed on March 26, 2013.

## RESULTS

After the procedure, the original nail was curetted, irrigated, and replaced. The nail remained in place for 10 days before falling off. Naftifine 2% cream was applied twice a day for 33 weeks. The nail required 33 weeks to regrow a length of 18 mm. The regrowth of the nail was photo-documented and compiled into a time-progression video viewable by following the link below.

## DISCUSSION

Nail avulsion is a common procedure indicated for both diagnostic and therapeutic reasons. It provides the surgeon access to the nail bed and the deeper germinal matrix, if a biopsy of either tissue is indicated. It is a treatment option with the most common indications being ingrown toenails, chronic onychomycosis, periungual warts, trauma, infections, and tumors. Avulsions can also occur as a result of injury rather than intention. In such cases, the degree and type of damage are the major factors that determine how the nail regrows. If the germinal matrix (which creates 90% of the nail plate) is damaged, then nail deformities are likely to occur.^5^^-^7 In a surgical avulsion, the germinal matrix and nail bed are left largely intact in order for the nail plate to regrow. To further avoid defect, the original nail or a prosthetic is positioned over the nail bed to inhibit synechial scarring of the ventral to dorsal germinal matrices and to protect the nail bed from infection.^8^^-^10

In the literature, nail regrowth has been documented under various circumstances. Following surgical avulsion, the combined range of regrowth for both fingernails and toenails has been observed at 5 to 10 months.^3^ Elsewhere it is estimated that surgically avulsed fingernails in the average adult will completely regrow in 4 to 5 months, whereas toenails require twice as long, 10 to 18 months.^4^^,^^11^ More precise rates of growth have been recorded in nonavulsed, healthy nails. One of the earliest studies reported growth rates of 0.094 to 0.124 mm/day, some variability explained by nutritional status.^12^ Another study on nail growth by Hamilton et. al recorded a range of growth rates 0.050 to 0.150 mm/day and found a significant difference between the growth rates of different fingers.^13^ Multiple other studies have reported growth rates since then with similar results.^14^^-^16

No study to date has compared normal nail growth rates with postavulsion growth rates. In our case, the patient's nail length regrew in approximately 33 weeks. The thumbnail length was 18 mm from the eponychium to the hyponychium. Therefore, the regrowth rate could be roughly approximated at 0.078 mm/day. A similar range of growth rates has been reported in nonavulsed nails. More precise studies need to be done to accurately compare growth between the 2 scenarios.

It is well known that there are numerous factors that affect the rate at which nails grow. The number and breadth of the factors identified explains why recorded growth rates are so variable. Reported factors include age, nutritional status, environmental temperature (faster growth in warm months), trauma, handedness (faster growth in dominant hand), pregnancy, acute illness, and preexisting skin conditions.^12^^-^15 It is well documented that the longitudinal nail growth rate slows with increasing age.^13^^,^^15^ Furthermore, the rates of growth vary in predictable patterns between the digits. The middle fingernail typically grows fastest, while the thumbnail and little fingernail have the slowest rates of growth.^13^^,^^14^

In our case, the patient was a 76-year-old man, nonsmoker with good nutritional status and no preexisting skin conditions. Therefore, it is plausible that the nail regrowth was at the lower end of reported ranges largely because of age, although growth of the thumbnail is slower independent of other factors.^13^^,^^14^ It is worth noting that Hamilton, et al found that nail thickness increases with age. Therefore, the nail may increase in total mass at the same rate regardless of age even though at older ages nail growth lengthwise slows.^13^

## CONCLUSIONS

Numerous, compounding factors affect nail regrowth, making the topic challenging to study. It is necessary to understand patterns of nail regrowth and the associated factors for the care and recovery of patients undergoing avulsion procedures. This information is helpful for explaining nail regrowth to patients. As such, further study still needs to be done to isolate the factors that particularly affect nail regrowth after surgical avulsion.
